# Supplementary material for: Hybrid Bioprinting for functionally graded tissue engineering constructs with patterned and localized biochemical signals
Source: Adv Compos Hybrid Mater. 2025 Nov 29;9(1):11. doi: 10.1007/s42114-025-01546-0 (PMC12783323; doi:10.1007/s42114-025-01546-0)
Supplement: Supplementary file 1 — Supplementary Material 1 [file 42114_2025_1546_MOESM1_ESM.docx]

**Hybrid Bioprinting for Functionally Graded Tissue Engineering Constructs with Patterned and Localized Biochemical Signals**

Jiannan Li^1†^, Carolyn Kim^1, 3†^, Hossein V. Alizadeh^1^, Shreya Garg^1, 2^, Arnaud Bruyas^1^, Peng Zhao^1^, Isadora S. D. Passos^1, 3^, Chi-Chun Pan^1^, Andrea S. Flores Pérez^1,2^, Mark A. Skylar-Scott^2^, Sungwoo Kim^1^, Yunzhi P. Yang^1, 2, 4^*

^1^Department of Orthopaedic Surgery, School of Medicine, Stanford University, Stanford, CA 94305, USA

^2^Department of Bioengineering, School of Engineering, Stanford University, Stanford, CA 94305, USA

^3^Department of Mechanical Engineering, School of Engineering, Stanford University, Stanford, CA 94305, USA

^4^Department of Material Science and Engineering, School of Engineering, Stanford University, Stanford, CA 94305, USA

^†^These authors contributed equally to this work.

^*^Corresponding author. Email: [ypyang@stanford.edu](mailto:ypyang@stanford.edu).

**Supplementary Information**

Figure S1. Working principle of Hybprinter-SAM.

Figure S2. Process flow of Hybprinting design and fabrication.

Figure S3. Pictures of Hybprinter-SAM assembly and working process.

Figure S4. Various demonstrations fabricated by Hybprinter-SAM.

Figure S5. Crosslinking mechanism of gelbrin hydrogel network.

Figure S6. Difference during 14-day culturing between cellular and acellular scaffolds.

Figure S7. Mechanical measurement setup for the hybprinted scaffold.

Figure S8. COMSOL simulation of temperature change in the hybprinted scaffold.

Figure S9. MTS proliferation study for 1 mm thick samples.

Figure S10. Fluorescence retention of patterned FITC-BSA onto SE printed gelbrin scaffold.

Figure S11. Design and setup of custom designed mechanical bioreactor.

Table S1. Important printing parameters for Hybprinter-SAM.

Table S2. Mechanical stimulus types and rates that can be provided by the custom designed bioreactor.

Table S3. Information of the primers used for qPCR.

Video SV1. Hybprinting process flow.

Video SV2. Hybprinted scaffold withstanding tension and compression.

Video SV3. Hybprinted scaffold withstanding torsion and bending.

Video SV4. Hybprinted scaffold with mesh hydrogel structure.

Video SV5. Flexibility of hybrid scaffold.

Video SV6. Smooth mechanical gradient created by crosslinker gradient.

Video SV7. Tensile test for hybrid scaffold.

Video SV8. Custom bioreactor for applying mechanical stretch.

Video SV9. Temperature change of PCL struts after printing.


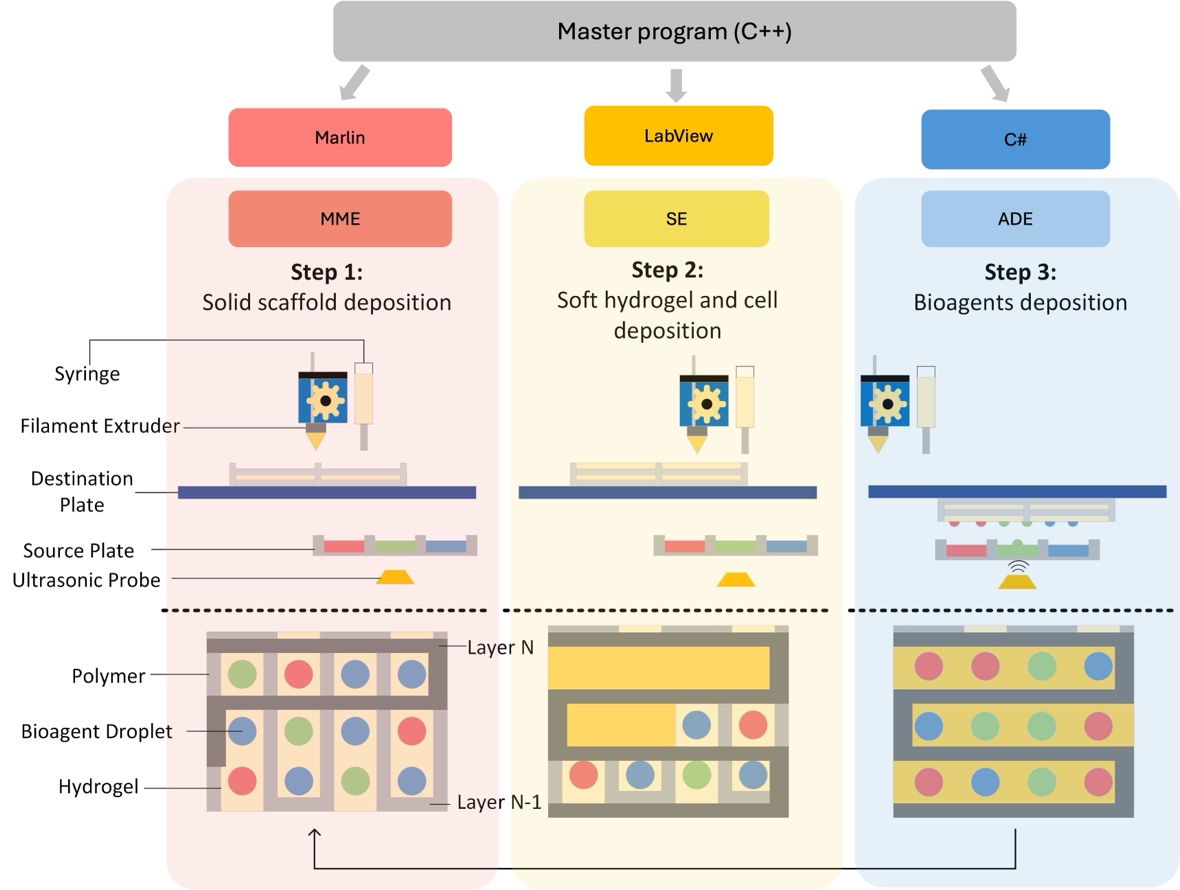


**Supplemental Figure S1.** Working principle of hybrid printing comprising molten material extrusion (MME), syringe extrusion (SE) and acoustic droplet ejection (ADE). All three modules are coordinated by custom developed software program, and they follow a layer-by-layer fabrication protocol in the order of MME🡪SE🡪ADE, resulting in an engineered construct comprising rigid polymeric scaffold, soft hydrogel scaffold, and aqueous-phase bioreagents.


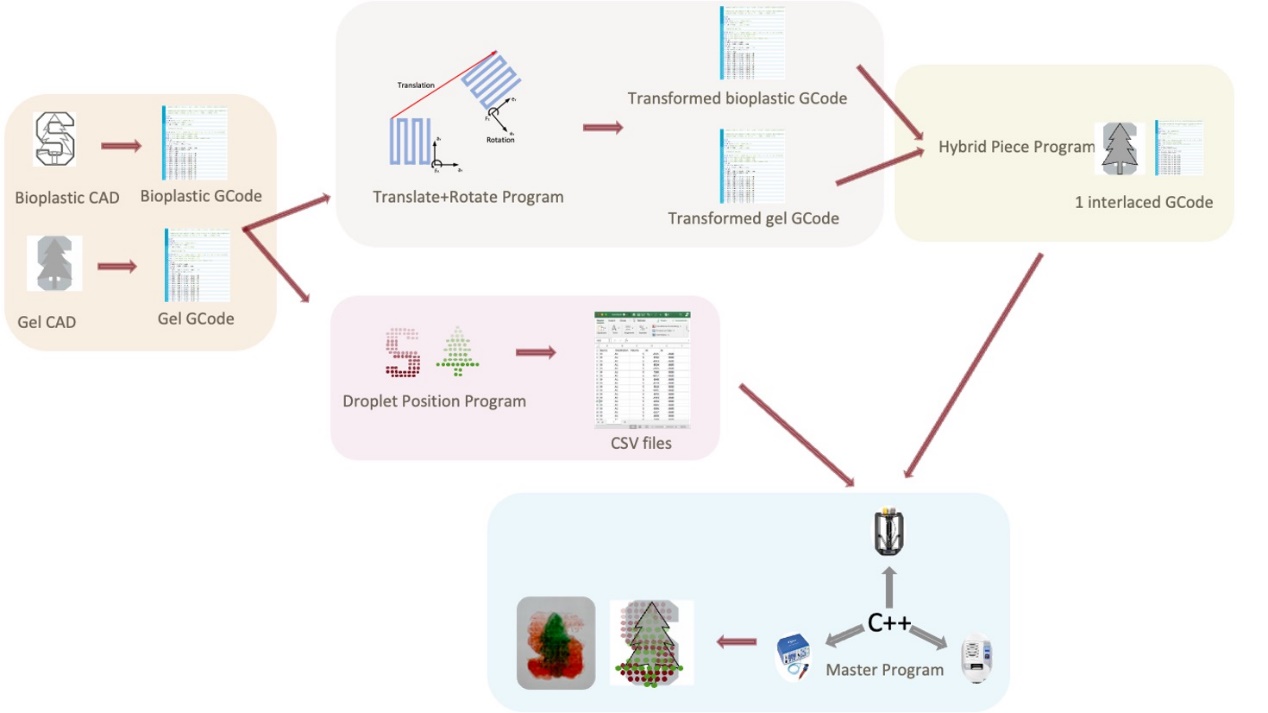


**Supplemental Figure S2.** Process flow of hybprinting design and fabrication including CAD design, slicing, coordinate system transformation, droplet pattern generation, and final master programming integration.


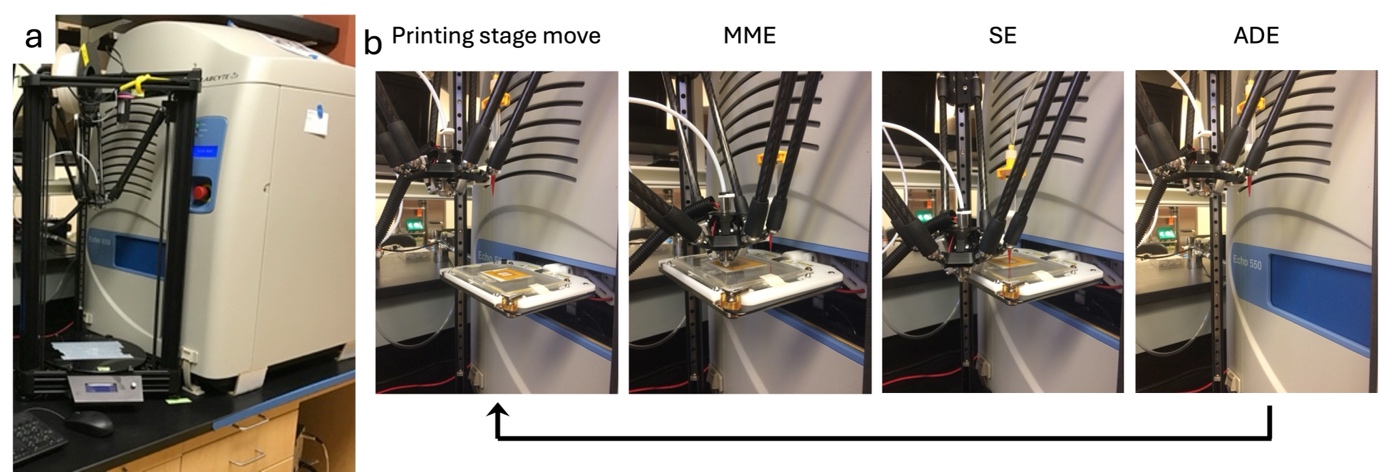


**Supplemental Figure S3.** (a) Assembly of Hybprinter-SAM. (b) Actual pictures showing process flow for Hybprinter-SAM, following a layer-by-layer fabrication protocol in the order of MME🡪SE🡪ADE.


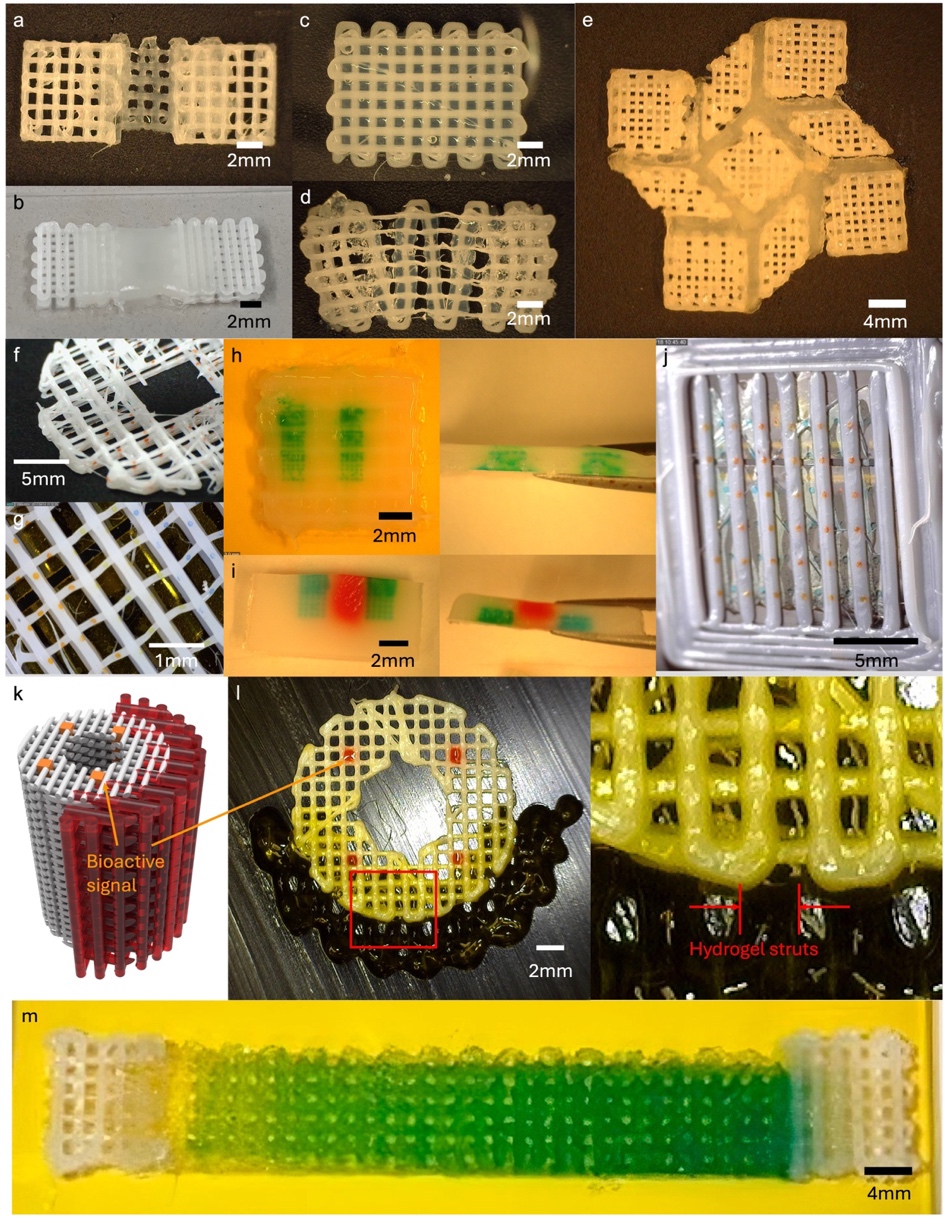


**Supplemental Figure S4.** (a-e) Hybrid soft-rigid tissue constructs of hydrogel and PCL that can withstand robust mechanical manipulation. (f, g) ADE deposition of droplets onto MME printed PCL struts. (h, i) Hydrogels with biological signal patterns. (j-l) Soft-rigid hybrid constructs with multiple biological factors (demonstrated by dyes) with different patterns, enabled by Hybprinter-SAM. (m) Printed sample of the soft-rigid construct with biological gradient, represented by color.


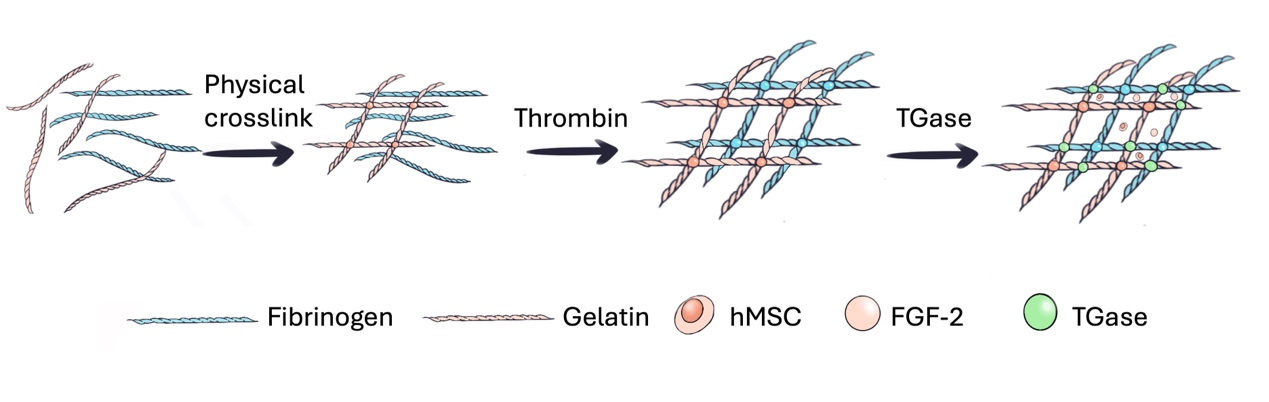


**Supplemental Figure S5.** Crosslinking mechanism of gelbrin hydrogel network: Gelatin first underwent temperature dependent physical crosslinking before printing; upon printing, thrombin first crosslinked fibrinogen into fibrin, followed by Ca^2+^-dependent TGase crosslinking of gelatin and fibrin.


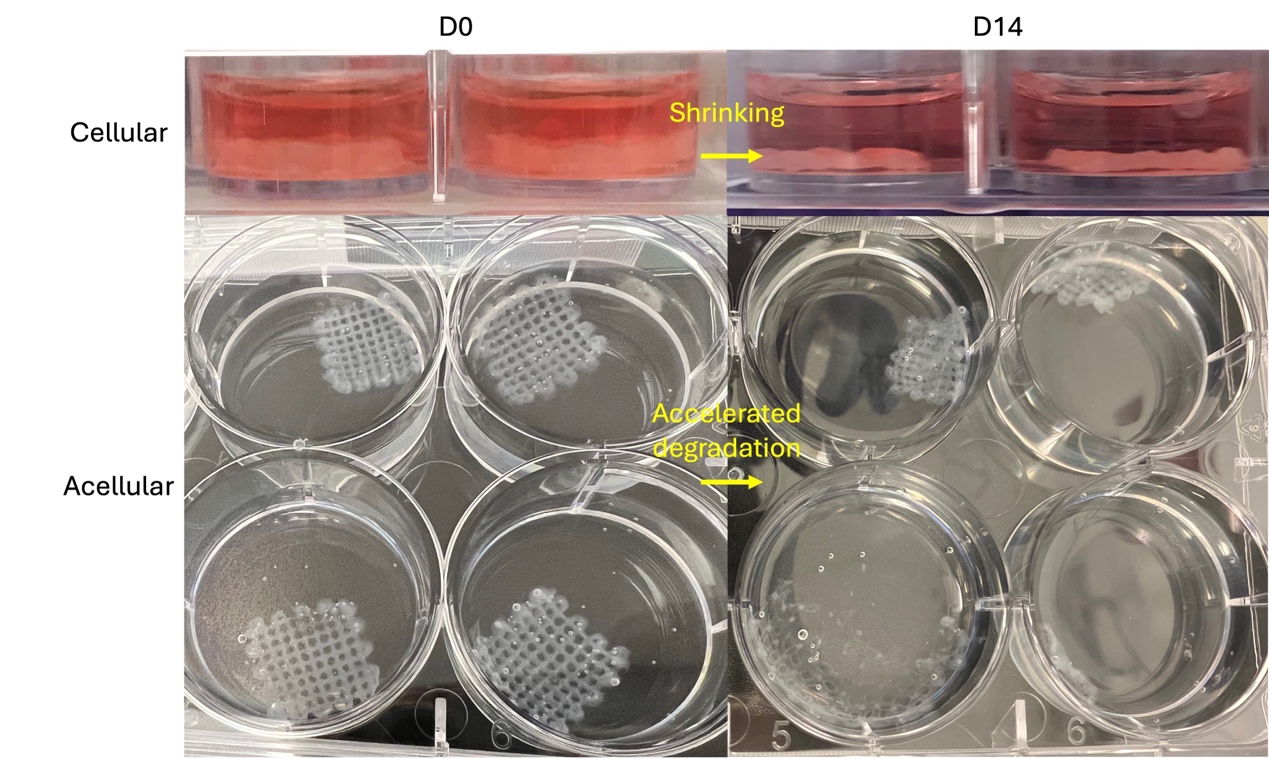


**Supplemental Figure S6.** Difference during 14-day culturing between cellular and acellular scaffolds, where cellular scaffolds shrank yet maintained its structural integrity, and acellular scaffolds almost fully degraded and dissembled.


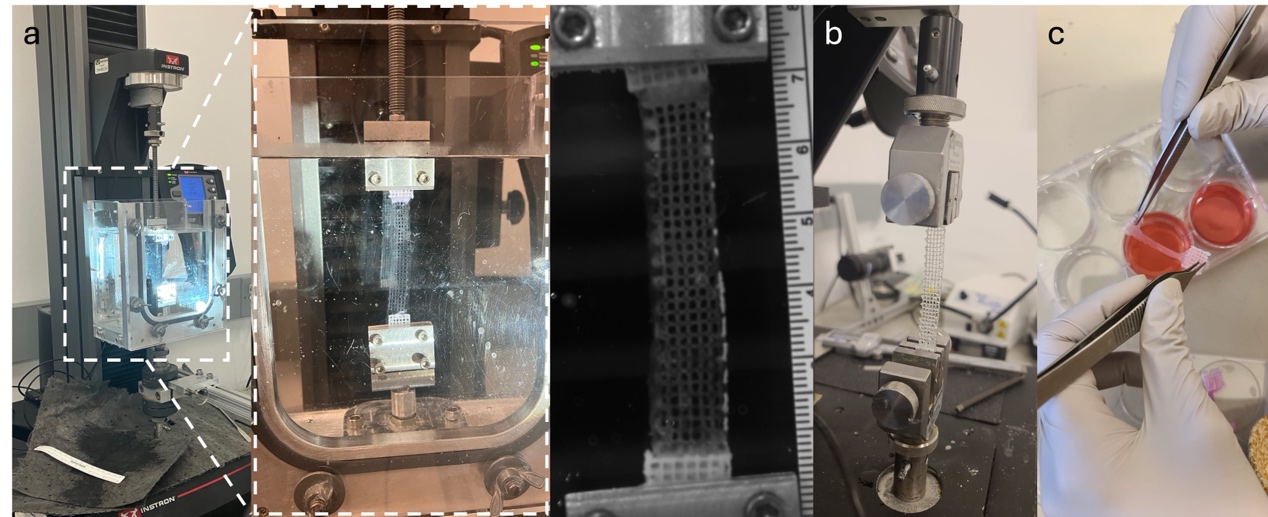


**Supplemental Figure S7.** Mechanical measurement setup for the hybprinted scaffold. (a) Measurement setup in water bath. (b) Measurement setup in air. (c) Gel deformation in air affecting measurement.


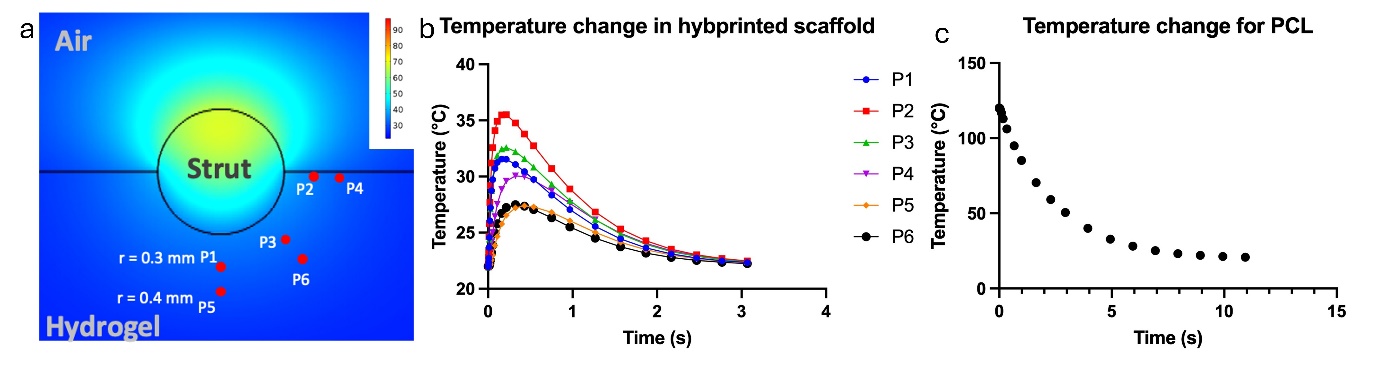


**Supplemental Figure S8.** COMSOL simulation of temperature change in the hybprinted scaffold. (a) Simulation design. (b) Simulation results for probes at different locations. (c) Temperature change of PCL struts after printed.


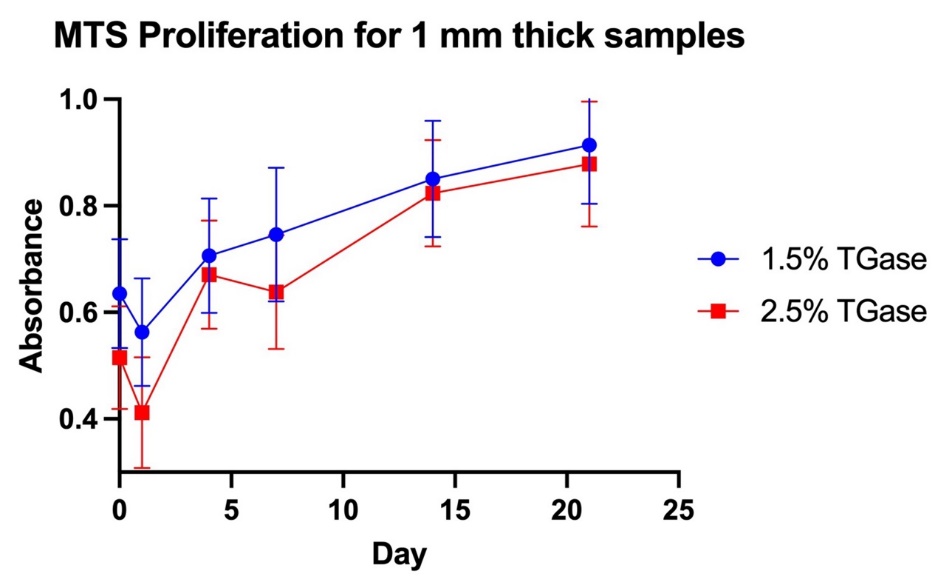


**Supplemental Figure S9.** MTS proliferation study for 1 mm thick samples.


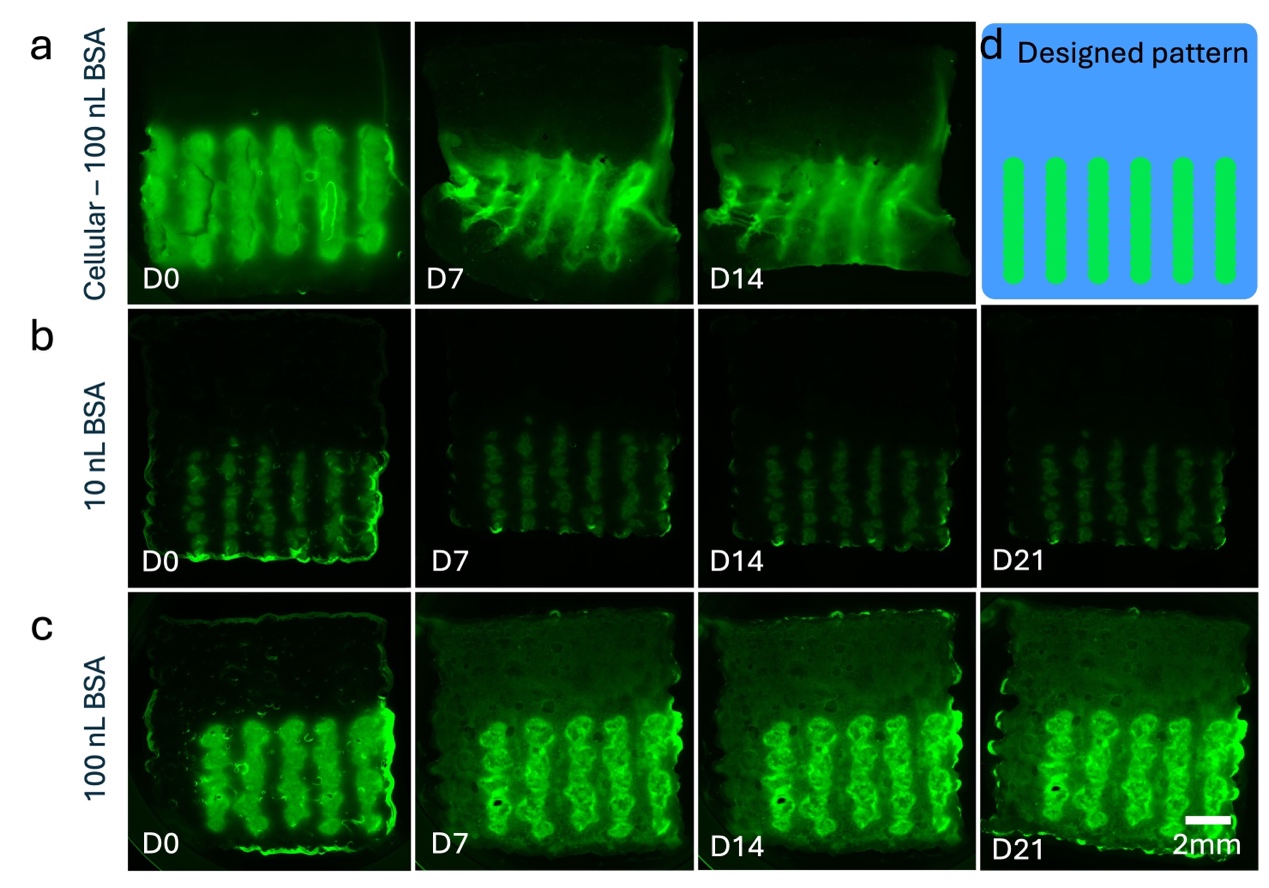


**Supplemental Figure S10.** (a-c) Fluorescence retention of patterned FITC-BSA onto SE printed gelbrin scaffold. (a) Cellular condition, 100 nL BSA droplet size. (b) Acellular condition, 10 nL BSA droplet size. (c) Acellular condition, 100 nL BSA droplet size. (d) Conceptual design of the pattern.


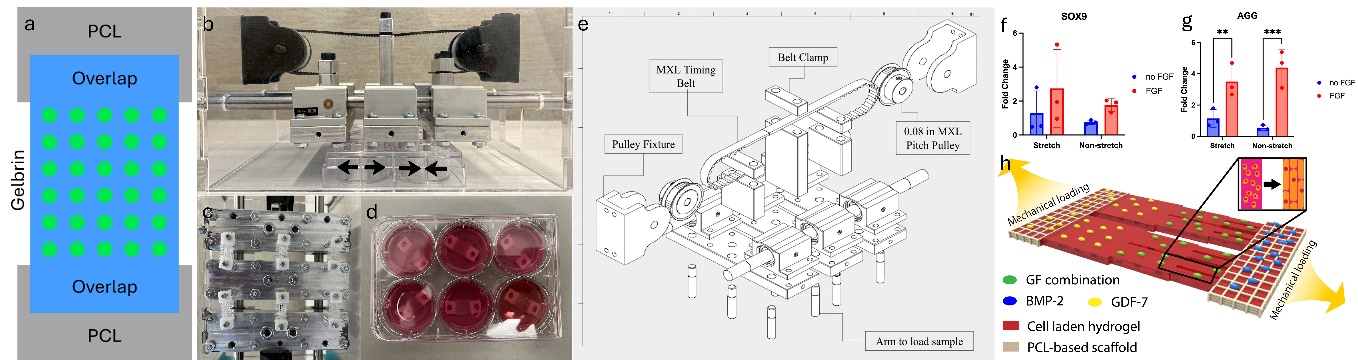


**Supplemental Figure S11.** (a) Schematic of printed pattern of FGF-2 where it’s only printed in the hydrogel region. (b) Side view of the bioreactor compatible with a 6-well plate. (c-d) Samples in a 6-well plate loaded onto the bioreactor. (e) An exploded schematic of the bioreactor’s hardware and working mechanism. (f-g) qPCR expression of (f) SOX9 and (g) AGG for comparison of mechanically stretched and non-stretched groups at D7. (h) Design for high-throughput screening comprising mechanical stimuli, soft-rigid material integration, combinatorial growth factors loading to study bone-tendon regeneration, where one may deposit BMP-2 for bone development, GDF-7 for tendon development, and screen different growth factors combinations for fibrocartilage development.


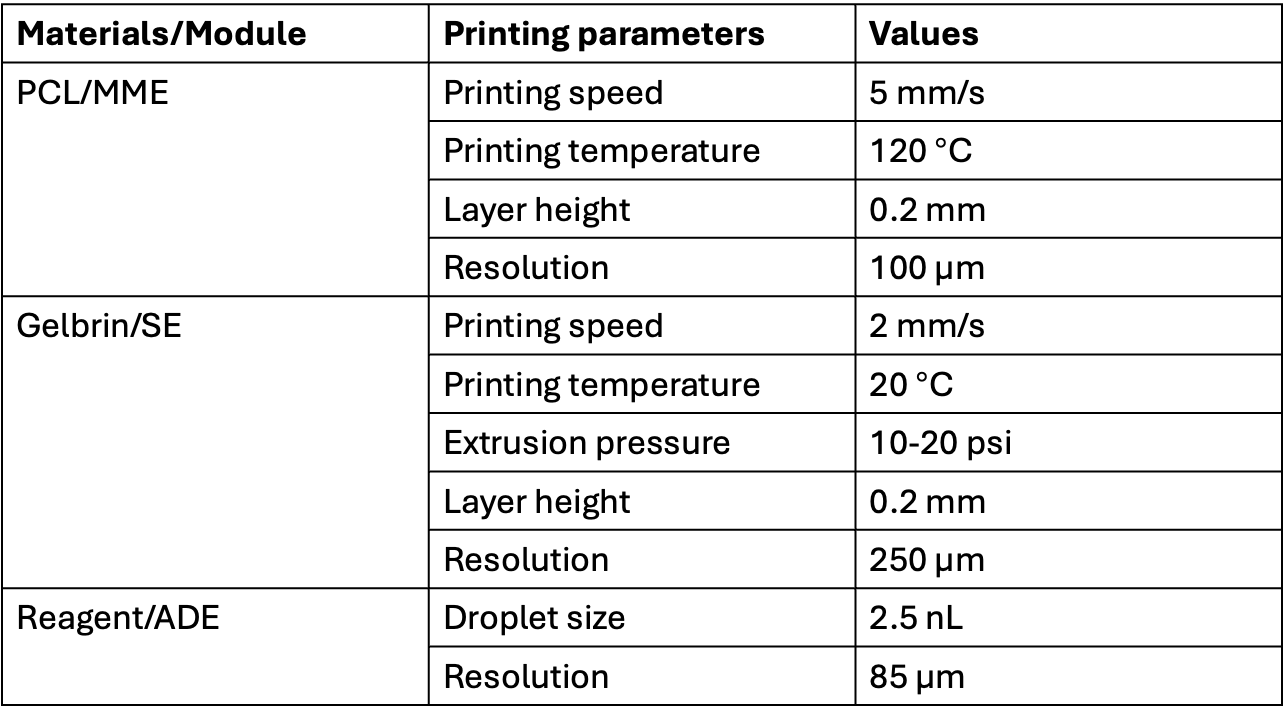


**Supplemental Table S1.** Important printing parameters for Hybprinter-SAM. Note that extrusion rate for PCL was a parameter set for controlling the actual strut size printed with MME.


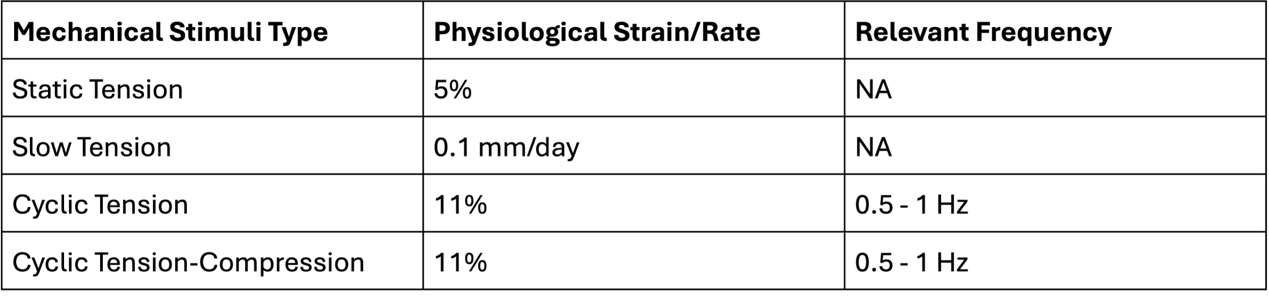


**Supplemental Table S2.** Mechanical stimulus types and rates that can be provided by the custom designed bioreactor.


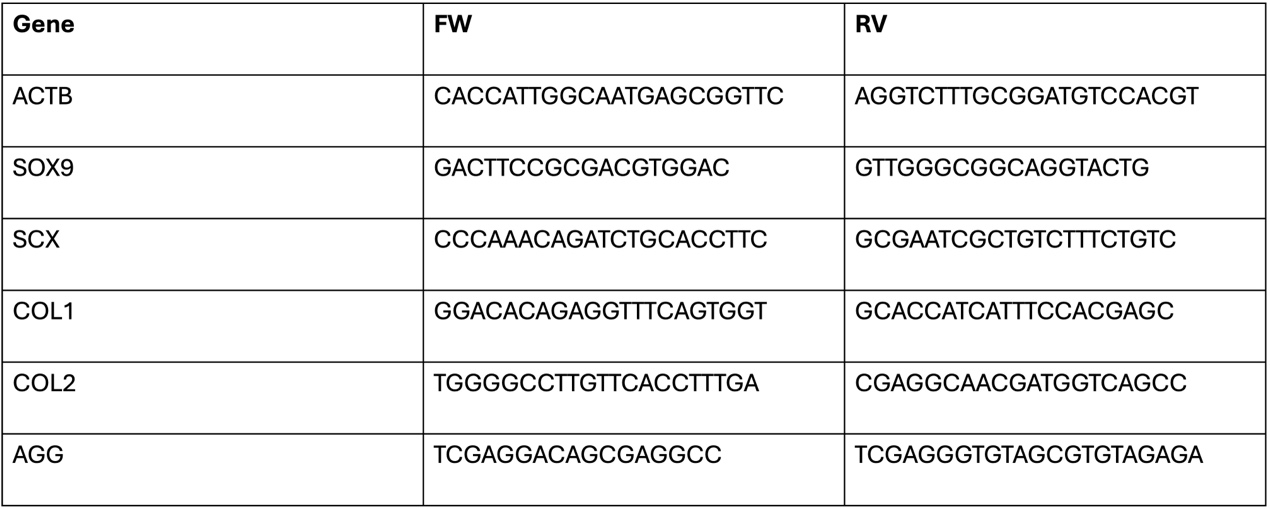


**Supplemental Table S3.** Information of the primers used for qPCR.
